# Irregular atrial arrhythmias shorter than 30 s and the risk of atrial fibrillation on continuous monitoring

**DOI:** 10.1093/europace/euaf300

**Published:** 2026-01-30

**Authors:** Nick L van Vreeswijk, Rajiv S Rama, Jeff S Healey, Emma Svennberg, Albin Edegran, Yuri Blaauw, Linda S Johnson, Michiel Rienstra

**Affiliations:** Department of Cardiology, University of Groningen, University Medical Center Groningen, Hanzeplein 1, 9713GZ Groningen, The Netherlands; Department of Cardiology, University of Groningen, University Medical Center Groningen, Hanzeplein 1, 9713GZ Groningen, The Netherlands; Population Health Research Institute, McMaster University, Hamilton, Ontario, Canada; Department of Medicine, McMaster University, Hamilton, Ontario, Canada; Karolinska Institutet, Department of Medicine Huddinge, Karolinska University Hospital, Stockholm, Sweden; Department of Clinical Sciences, Malmö. Lund University, Lund, Sweden; Department of Cardiology, University of Groningen, University Medical Center Groningen, Hanzeplein 1, 9713GZ Groningen, The Netherlands; Department of Clinical Sciences, Malmö. Lund University, Lund, Sweden; Department of Cardiology, University of Groningen, University Medical Center Groningen, Hanzeplein 1, 9713GZ Groningen, The Netherlands

**Keywords:** Atrial Fibrillation, Ambulatory ECG monitoring, Screening, Stroke prevention

## Introduction

Atrial fibrillation (AF) is associated with increased stroke risk, which can be mitigated with oral anticoagulation (OAC).^1–3^ The risk of stroke is lower among patients with low burden AF, but in patients with high CHA_2_DS_2_-VA scores or a previous stroke the benefits of OAC treatment outweigh bleeding risks.^4,5^ According to the 2024 European Society of Cardiology guidelines, a clinical diagnosis of AF can be made with either 12-lead ECG or ≥30 s of AF on an ambulatory ECG recording.^6^ However, shorter episodes of irregular atrial arrhythmias that do not meet the duration requirement commonly occur, and these have been shown to be associated with hospitalization for AF in observational studies.^7,8^ The appropriate way to manage these arrhythmias in clinical practice is currently unknown.^9^

This study investigates whether irregular atrial arrhythmias lasting <30 s that are detected during the first 48 h of ambulatory ECG monitoring are associated with increased occurrence of AF with ≥30 s duration during subsequent monitoring for up to 30 days.

## Methods

**Figure 1:**
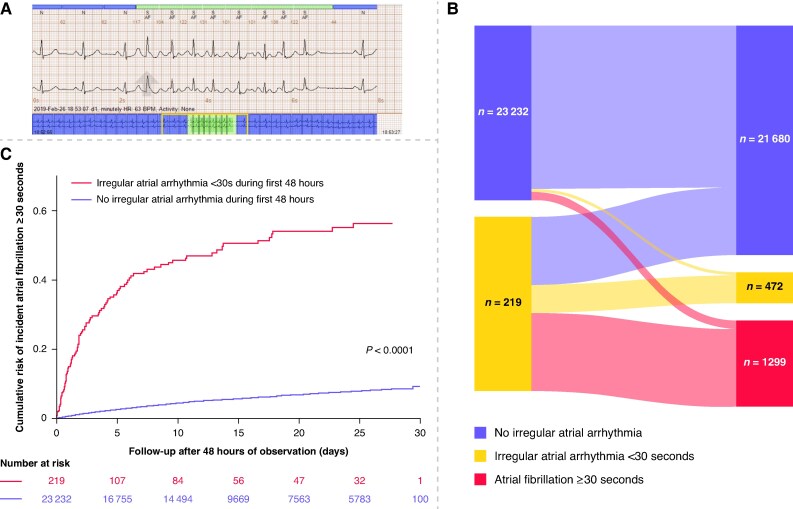
(*A*)**: Representative ECG recording of an irregular atrial arrhythmia <30 s -** the rhythm strip shows a sudden onset of rapid, irregular atrial activity without discernible P-waves, followed by spontaneous termination and return to sinus rhythm. The episode lasted less than 30 s and therefore does not fulfil the diagnostic criterion for clinical atrial fibrillation. (*B*) **Progression of atrial arrhythmia <30 s during extended ECG monitoring -** Proportions of 219 patients with atrial arrhythmia <30 s during the first 48 h of ambulatory ECG monitoring who subsequently had atrial fibrillation episodes ≥30 s (n = 100), additional episodes <30 s (n = 34), or no further atrial arrhythmias during the remaining follow-up period (n = 85). These results highlight the heterogeneity of short atrial arrhythmias and their varying likelihood of progression to clinically defined AF. *Note: For visualization purposes, the two baseline groups are displayed with equal width in the Sankey diagram, although only 219 of 23 451 patients had atrial arrhythmias during the first 48 h.* (*C*) **Cumulative risk of incident AF ≥30 s during extended monitoring -** Kaplan–Meier curves showing the cumulative incidence of AF episodes ≥30 s during extended follow-up, stratified by the presence or absence of irregular atrial arrhythmia <30 s during the first 48 h of monitoring. Patients with irregular atrial arrhythmia <30 s in the first 48 h had a significantly higher incidence of subsequent AF episodes compared to those without (adjusted HR 8.28, 95% CI 6.74–10.19, p < 0.001).

We analysed 30-day ambulatory ECG monitor data from 32 146 patients monitored for a clinical indication in the United States in 2021, after referral from both primary and tertiary care centres. The ECG signals were collected using the PocketECG system (MEDICALgorithmics, Warsaw, Poland), a device that records and transmits full-disclosure continuous ECG signals with a limb lead configuration (leads II and III) and a sampling rate of 300 Hz, for up to 31 days. The signals were analysed using an FDA approved algorithm (MEDICALgorithmics, Warsaw, Poland) capable of detecting irregular atrial episodes lasting ≥4 beats. All detected arrhythmia events were manually verified and corrected by a licensed ECG technician in clinical practice. An example of an irregular atrial arrhythmia lasting <30 s can be seen in *Figure 1A*.

We excluded patients with <48 h of recordings (*n* = 6741) or AF episodes ≥30 s in the first 48 h of monitoring (*n* = 1954). Irregular atrial arrhythmias <30 s were defined as any irregular supraventricular arrhythmias without discernible *P*-waves, that would have been considered AF if the duration had exceeded 30 s. The association between <30 s irregular atrial arrhythmias and AF ≥ 30 s during the subsequent ≤30 days of registration was analysed using age- and sex- adjusted Cox regression. *P*-values <0.05 denote statistical significance.

Monitoring indication was collected on device connection, and we conducted a sensitivity analysis in the patients whose ambulatory ECG monitoring indication was suspected atrial arrhythmia, including monitoring for palpitations or AF, atrial flutter, or atrial tachycardia. All analyses were conducted in R version 4.5.1 (released June 2025).

## Results

The final study population consisted of 23 451 individuals, of whom 60.9% were female. The median age was 61 years [interquartile range (IQR) 45–72 years]. The most common indication for monitoring was palpitations or for detection of AF or other supraventricular arrhythmias (*n* = 15,630, 66.6%). Monitoring indications also included syncope or presyncope (*n* = 2,650, 11.3%), stroke or transient ischaemic attack (TIA) (*n* = 1,462, 6.2%) and other indications, including angina pectoris, conduction disorders, and ventricular arrhythmias (*n* = 3,709, 15.8%).

Irregular atrial arrhythmias <30 s were detected in 219 individuals (0.93%) within the first 48 h. The median episode duration was 6.6 s (IQR 1.8–15.9 s). Compared to patients without irregular atrial arrhythmias <30 s during the first 48 h, these patients were older (median age 73 vs. 61 years, *P* < 0.001) and more frequently male (48.9% vs. 39.0%, *P* = 0.004).

The median recording time was 11.9 days (IQR 4.8–25.4), during which 1299 patients (5.5%) had episodes of AF ≥30 s. Patients with irregular atrial arrhythmias <30 s during the initial 48 h had a high probability of additional arrhythmia during prolonged monitoring; 100 (45.7%) individuals subsequently had AF episodes ≥30 s and 34 (15.5%) had additional irregular atrial arrhythmia episodes <30 s, (*P* < 0.001, *Figure 1B*). In patients with irregular atrial arrhythmias <30 s who subsequently had ≥30 s AF (*n* = 100), the maximum AF episode duration was in median 25.7 min (IQR: 1.9–223.7, range: 0.5–15 748.6 min), and the median AF burden during follow-up was 0.57% of the monitored time (IQR: 0.06–3.25%, range: 0.04% to 98.18%). Of patients without any irregular atrial arrhythmia during the first 48 h (*n* = 23 232), only 1199 (5,2%) progressed to AF ≥30 s.

After adjustment for age and sex, irregular arrhythmia episodes <30 s were independently associated with a substantially increased probability of AF ≥ 30 s (hazard ratio [HR] 8.28, 95% confidence interval [CI] 6.74–10.19, *P* < 0.001, *Figure 1C*). Similar results were found in the sensitivity analysis restricted to patients monitored to detect atrial arrhythmias, adjusted HR 6.88, 95% CI 5.40–8.75, *P* < 0.001.

## Discussion

Irregular atrial arrhythmias <30 s were present in a minority of patients in the first 48 h of ECG recordings, but half of these subsequently had AF episodes ≥30 s during extended monitoring. In patients who have been monitored for a short time period in which an irregular atrial arrhythmia <30 s has occurred, extended monitoring should be considered if the patient has sufficient stroke risk. Based on findings in the ARTESiA and NOAH-AFNET 6 trials, this could include patients with a high CHA₂DS₂-VA score,^5^ vascular disease^10^ or a prior stroke.^4^

The strengths of our study include the large sample size and prolonged monitoring durations. However, a key limitation is the lack of clinical data on individual patients, limiting our ability to assess the stroke risk of individuals with irregular atrial arrhythmias <30 s who progress to longer AF episodes. This limitation also hampers interpretation of the low incidence (<1%) of short irregular atrial arrhythmias, which may in part reflect the characteristics of the monitored cohort rather than the prevalence in an unselected, real-world population.

Patients with AF ≥30 s in the first 48 h, who may also have had shorter episodes, were excluded, likely contributing to underestimation of true frequency.

## Conclusion

Nearly half of patients with AF-like atrial arrhythmia <30 s on initial ECG monitoring progressed to AF ≥30 s during extended follow-up. These findings support prolonged monitoring in patients with high risk of stroke, such as patients with a high CHA₂DS₂-VA score, vascular disease or with a prior stroke.

## Data Availability

The data that supports the findings of this study are derived from patient ECGs and are not publicly available due to privacy concerns but will be made available after a request for access to the corresponding author for the purpose of reviewing the study results and at the cost of a data preparation fee. No requests that include a commercial interest will be approved. Data are located in controlled access data storage at MEDICALgorithmics. A response to a request to access the data can be expected within 2 months.
